# Left ventricular ejection fraction as an independent predictor of poor outcome in acute intracerebral hemorrhage

**DOI:** 10.1002/brb3.1643

**Published:** 2020-06-09

**Authors:** Guang Yang, Lu Wang, Jingxian Sun, Daming Zhang, Ruotian Zhang, Chao Yuan, Meixin Long, Yingqiang Zhong, Chunmei Li, Xiaoxiong Wang, Xin Chen, Qi Zhou, Bo Liu, Hongquan Jiang, Ailing Lian, Ilgiz Gareev, Lili Li, Shiguang Zhao

**Affiliations:** ^1^ Department of Neurosurgery The First Affiliated Hospital of Harbin Medical University Harbin China; ^2^ Institute of Brain Science Harbin Medical University Harbin China; ^3^ Department of Urology The Fourth Hospital of Harbin Medical University Harbin China; ^4^ Section of Surgical Outcomes and Epidemiology Department of Surgery Yale School of Medicine New Haven Connecticut; ^5^ Department of Urology The First Affiliated Hospital of Harbin Medical University Harbin China; ^6^ Department of Neurology The First Affiliated Hospital of Harbin Medical University Harbin China; ^7^ Department of Neurology The Fourth Hospital of Harbin Medical University Harbin China; ^8^ Research Administration Office The First Affiliated Hospital of Harbin Medical University Harbin China; ^9^ Operating Room The First Hospital of Harbin Medical University Harbin China; ^10^ Bashkir State Medical University Ufa Russia

**Keywords:** acute intracerebral hemorrhage, left ventricular ejection fraction, outcome, predictor

## Abstract

**Introduction:**

Few studies of the effect of cardiac abnormalities on acute intracerebral hemorrhage (ICH) outcomes have been published. We sought to determine whether the left ventricular ejection fraction (LVEF) is associated with the functional outcome and mortality of acute ICH patients.

**Methods:**

We conducted a retrospective study on 364 acute ICH patients from January to December 2016. The primary outcome was defined by the modified Rankin Scale and mortality at 3 months. The associations between LVEF and outcome were investigated using univariable and multivariable logistic regression models.

**Results:**

Depressed LVEF was significantly associated with a poor functional outcome with an odds ratio [OR] of 0.966, 95% confidence interval (CI) 0.942–0.991, *p* = .008, and high mortality (OR 0.968 [95% CI 0.943–0.994], *p* = .015) at 3 months for acute ICH patients by univariate analysis. Multivariable logistic regression analysis indicated that LVEF was an independent predictor of a poor functional outcome (OR 0.961 [95% CI 0.935–0.988], *p* = .005) and mortality (OR 0.949 [95% CI 0.918–0.981], *p* = .002). The percentage of acute ICH patients with poor functional outcome (*p* = .005) and mortality (*p* = .002) was obviously higher in the group of patients with a LVEF of <50%.

**Conclusions:**

LVEF is an independent predictor of functional outcome and mortality at 3 months for acute ICH patients. These findings could provide the evidence needed for prognosis prediction in acute ICH patients.

## Graphical abstract

Left ventricular ejection fraction is an independent predictor of functional outcome and mortality at 3 months for acute intracerebral hemorrhage (ICH) patients. These findings could provide the evidence needed for prognosis prediction in acute ICH patients.

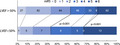

## 1 INTRODUCTION

Acute intracerebral hemorrhage (ICH) is a major cause of death and disability with few effective treatment options (James et al., 2017). Previous studies have shown that some of the patients with intracerebral hemorrhage suffer an acute serious cardiac complication (Adelborg et al., 2017; Putaala et al., 2014). The left ventricular ejection fraction (LVEF) is a widely accepted clinical indicator of left ventricular systolic function and a risk factor for stroke events in heart failure patients (Di Tullio et al., 2016). Although few studies have revealed that LVEF can also impact neurological outcomes in ischemic stroke, it has not been reported in ICH (Milionis et al., 2013; Pana et al., 2019). The primary aim of this study was to evaluate the predictive value of LVEF on the functional outcome and mortality of patients with acute ICH.

## 2 MATERIALS AND METHODS

### 2.1 Patient characteristics

Patients aged ≥18 years presenting to the First Affiliated Hospital of Harbin Medical University with acute ICH who were admitted within 3 days of onset between January and December 2016 were enrolled in an observational cohort study. Patients with ICH attributed to trauma, hemorrhagic conversion of ischemic stroke, structural lesions, or vascular malformations were excluded. Demographic and clinical data were systematically collected through interviews with patients, family members, and a retrospective review of the hospital's medical records. The Glasgow Coma Scale (GCS) score was prospectively recorded at the time of initial evaluation by a trained neurologist or neurosurgeon. All patients had sinus rhythm, and patients with severely depressed LVEF (<35%) and cardiomyopathy were excluded. The study was approved by the Institutional Review Board of the First Affiliated Hospital of Harbin Medical University, and the participants gave informed consent.

### 2.2 Calculation of hematoma volume

In all patients, a brain CT scan was obtained within 24 hr of admission. CT image data sets were acquired on the standard Digital Imaging and Communications in Medicine (DICOM) format and then assessed with 3D Slicer 3.6.1 open source software (SPL, Harvard Medical School). Hematomas were automatically identified pixel by pixel in each slice, and a 3D model was constructed. The hematoma volume was calculated by the accumulating volume of the pixels.

### 2.3 LVEF determination

All patients underwent a comprehensive Doppler echocardiography within 24 hr of admission by an attending cardiologist who was blinded to the clinical status of the patient. Standard parasternal long‐axis, short‐axis, and apical 2‐ and 4‐chamber views were obtained for analysis of LV dysfunction. All echocardiograms included at least five cardiac cycles and were digitally stored. LVEF was measured from the apical four‐ and two‐chamber views, using the standard biplane Simpson's rule.

### 2.4 Follow‐up and outcome events

Follow‐up is assessed at a structured telephone interview by a neurosurgeon. The modified Rankin Scale (mRS) was used to evaluate the functional outcome after 3 months. Poor functional outcome was defined as a mRS score of 3–6, and a favorable outcome was defined as a mRS score of 0–2.

### 2.5 Statistical analysis

Statistical analysis was performed using SPSS for Mac (version 21.0, IBM Corp). Categorical variables were expressed as counts (percentages), whereas continuous variables were expressed as mean ± *SD* or median (interquartile range [IQR]) values. The differences between patients with LVEF higher or lower than 50% were examined using the chi‐square test, Student's *t*‐test, or Mann–Whitney *U* test as appropriate. Correlations between continuous variables were assessed by Pearson's correlation or Spearman's correlation coefficients depending on data normality, and the association between LVEF and heart rate/systolic blood pressure was examined using a linear regression. Multiple logistic regression was calculated to identify predictors for poor functional outcome and mortality after incorporating the variables associated with mortality and poor outcome after 3 months in the univariate analyses (*p* < .05). Statistical significance was defined as *p* < .05 for all tests.

## 3 RESULTS

Among the 398 patients with primary ICH, 15 missed available follow‐up information, 19 were excluded due to LVEF < 35%, and 364 were included in our study. Detailed baseline characteristics are displayed in Table 1. Median LVEF was 61% (58%–63%). Our analysis showed no relationship between LVEF and on‐admission systolic blood pressure (BP; *ρ* = −0.015; *p* = .770; Figure S1) or heart rate (*ρ* = −0.045; *p* = .388; Figure S2).

**TABLE 1:** Baseline characteristics

Characteristics	*n* = 364
Age, years	61.1 ± 13.4
Sex (male)	240 (65.9)
History of hypertension	252 (69.2)
History of diabetes	61 (16.8)
CAD	60 (16.5)
History of atrial fibrillation	31 (8.5)
First systolic BP, mm Hg	177.9 ± 32.2
Heart rate	84.1 ± 20.4
Temperature, °C	36.6 ± 0.5
Admit GCS	13 (9–15)
Admit NIHSS	10 (4–18)
Anticoagulant	65 (17.9)
Antiplatelet	78 (21.4)
Initial hematoma volume, ml	13.8 (5.8–31.7)
IVH	127 (34.9)
Location of ICH	
Lobar	48 (13.2)
Deep	235 (64.6)
Infratentorial	40 (11.0)
Midline shift, mm	3.5 ± 3.8
Admission HCT, %	41.9 (38.4–45.3)
Admission platelet count, K/µl	222.7 (184.4–264.9)
Admission INR	1.1 (1.0–1.1)
Glucose, mmol/L	7.1 (5.9–9.4)
LVEF, %	61 (58–63)
Surgery	103 (28.3)

Note

Values are *n* (%), mean ± *SD*, or median (interquartile range).

Abbreviations: BP, blood pressure; CAD, coronary artery disease; GCS, Glasgow Coma Scale; HCT, hematocrit; ICH, intracerebral hemorrhage; INR, international normalized ratio; IVH, intraventricular hemorrhage on presentation; LVEF, left ventricular ejection fraction; mRS, modified Rankin Scale; NIHSS, NIH Stroke Scale.

Table 2 shows the results of univariate logistic regression analysis of factors associated with functional outcome and mortality at 3 months. Univariate analysis demonstrated that the LVEF was significantly associated with a poor functional outcome (OR 0.966 [95% CI 0.942–0.991], *p* = .008) and high mortality (odds ratio [OR] 0.968 [95% CI 0.943–0.994], *p* = .015). In multivariable logistic regression analysis, LVEF remained an independent predictor of poor functional outcome (OR 0.961 [95% CI 0.935–0.988], *p* = .005) and high mortality (OR 0.949 [95% CI 0.918–0.981], *p* = .002), after adjustment for the other covariates that were marginally or significantly associated with poor functional outcome and mortality in univariate analysis (Table 3).

**TABLE 2:** Univariate associations with poor functional outcome (mRS, 3–6)

Variable	3‐month Poor outcome			3‐month Mortality		
	OR	95%‐CI	*p* Value	OR	95%‐CI	*p* Value
Age, years	1.018	1.002–1.034	.030	1.018	0.998–1.037	.077
Sex (male)	1.018	0.660–1.570	.936	0.960	0.560–1.643	.881
History of hypertension	0.846	0.542–1.321	.463	0.514	0.304–0.870	.013
History of diabetes	0.799	0.460–1.390	.428	0.622	0.291–1.327	.219
History of atrial fibrillation	1.733	0.815–3.683	.153	1.381	0.591–3.224	.456
CAD	1.054	0.606–1.834	.852	1.356	0.708–2.594	.358
First systolic BP, mm Hg	1.006	1.000–1.013	.056	1.004	0.996–1.012	.279
Heart rate	1.011	1.001–1.022	.033	1.026	1.014–1.039	<.001
Temperature	1.367	0.857–2.178	.189	1.455	0.894–2.368	.132
Admit GCS	0.824	0.775–0.876	<.001	0.780	0.730–0.832	<.001
Admit NIHSS	1.081	1.052–1.110	<.001	1.081	1.049–1.114	<.001
Anticoagulant	0.645	0.374–1.113	.115	0.748	0.370–1.513	.419
Antiplatelet	0.762	0.460–1.261	.290	0.897	0.477–1.685	.735
Initial hematoma volume, ml	1.013	1.005–1.022	.003	1.015	1.006–1.024	.001
IVH	1.637	1.044–2.567	.032	1.348	0.770–2.357	.296
Location of ICH	1.368	0.896–2.091	.147	1.507	0.880–2.580	.135
Midline shift, mm	1.047	0.987–1.109	.125	1.046	0.978–1.118	.192
Admission HCT, %	0.956	0.919–0.994	.025	0.956	0.912–1.003	.065
Admission platelet count, K/µl	1.000	0.998–1.003	.776	1.000	0.996–1.003	.964
Glucose, mmol/L	1.067	1.001–1.136	.046	1.115	1.041–1.194	.002
Admission INR	0.969	0.489–1.921	.928	1.540	0.765–3.103	.227
LVEF, %	0.966	0.942–0.991	.008	0.968	0.943–0.994	.015
Surgery	1.358	0.859–2.145	.191	0.982	0.558–1.728	.949

Abbreviations: BP, blood pressure; CAD, coronary artery disease; CI, confidence interval; GCS, Glasgow Coma Scale; HCT, hematocrit; ICH, intracerebral hemorrhage; INR, international normalized ratio; IVH, intraventricular hemorrhage on presentation; LVEF, left ventricular ejection fraction; mRS, modified Rankin Scale; NIHSS, NIH Stroke Scale; OR, odds ratio.

**TABLE 3:** Multiple associations with poor functional outcome (mRS, 3–6)

Variable	3‐month Poor outcome			3‐month Mortality		
	Adjusted OR	95%‐CI	*p* Value	Adjusted OR	95%‐CI	*p* Value
Age, years	1.014	0.995–1.033	.160	1.020	0.995–1.046	.123
History of hypertension	0.847	0.500–1.435	.538	0.426	0.216–0.838	.013
Heart rate	0.998	0.985–1.011	.771	1.016	1.000–1.032	.055
Admit GCS	0.840	0.786–0.897	<.001	0.780	0.723–0.843	<.001
Admit NIHSS	1.021	0.979–1.066	.324	0.983	0.932–1.037	.537
Initial hematoma volume, ml	1.001	0.991–1.011	.865	1.001	0.990–1.012	.866
Admission HCT, %	0.164	0.001–21.329	.467	0.014	0.000–7.193	.181
Glucose, mmol/L	0.990	0.917–1.070	.808	1.014	0.924–1.114	.765
LVEF, %	0.961	0.935–0.988	.005	0.949	0.918–0.981	.002

Abbreviations: CI, confidence interval; GCS, Glasgow Coma Scale; HCT, hematocrit; LVEF, left ventricular ejection fraction; NIHSS, NIH Stroke Scale; OR, odds ratio.

**FIGURE 1:**
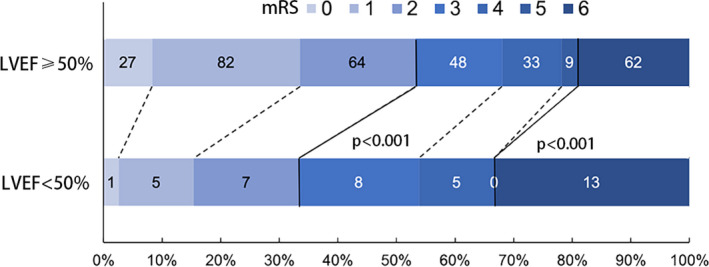
Distribution of the modified Rankin Scale (mRS) according to 50% LVEF. The bold line separates favorable (mRS, 0–2) and poor outcome (mRS, 3–6), or survival and death

In order to facilitate clinical work, LVEF was artificially divided into LVEF ≥ 50% or LVEF < 50%. The proportion of patients with poor functional outcome and mortality at 3 months was significantly higher in patients with an LVEF of <50% (mRS, 3–6 at 3 months; LVEF < 50%, 26/39 [66.7%] vs. LVEF ≥ 50%, 152/325 [46.8%], *p* = .005; mortality at 3 months: LVEF < 50% 13/39 [33.3%] vs. LVEF ≥ 50% 62/325 [19.1%], *p* = .002; Figure 1). Demographics and clinical characteristics of the cohort by LVEF category are shown in Table S1.

## 4 DISCUSSION

In this study cohort of 364 acute ICH patients, we show a significant and independent relationship between functional neurological outcome and mortality at 3 months based on the LVEF for these patients. Depressed LVEF was significantly associated with a poor functional outcome and high mortality. When we divided the LVEF by 50%, the patients with a lower LVEF had more poor neurological functional outcome and mortality.

Currently, there are no reports on the prognosis of ejection fraction (EF) in ICH, but the studies have shown that acute ischemic stroke patients with low EF were associated with a high risk of early and long‐term functional disability and mortality (Milionis et al., 2013). Similarly, severe left ventricular dysfunction after subarachnoid hemorrhage (SAH) can have an increase in risk of neurogenic cardiac complications, such as cerebral infarction, due to hypotension and vasospasm (Temes et al., 2010). In our study, the results firstly showed that ICH patients with low LVEF have worse neurological function outcomes and higher risks of mortality at 3 months. Furthermore, heart rate and systolic BP have been reported to be independently associated with poor functional outcome and mortality after acute ICH (Qiu et al., 2016; Qureshi et al., 2016). Higher admission heart rate also showed to be associated with poor functional outcome and mortality, but no relationship between LVEF and on‐admission systolic blood pressure in our study.

This study has several shortcomings. Firstly, this was a retrospective study conducted on Chinese patients from one hospital. Prospective, multicenter trials are needed to confirm similar findings in other hospitals, ethnic groups, and mixed populations. Secondly, this study does not measure the intracranial pressure or cerebral blood flow in ICH patients. The intracranial pressure or cerebral blood flow is a key factor for ICH patients’ outcome and mortality.

## 5 CONCLUSIONS

Elevated LVEF is significantly associated with a favorable functional outcome and low mortality, and LVEF is an independent predictor of functional outcome and mortality at 3 months for acute ICH patients.

## CONFLICT OF INTEREST

The authors have no conflict of interest to declare.

## AUTHOR CONTRIBUTIONS

GY, LW, and JS conceived and designed the study. RZ, CY, ML, YZ, CL, XC, XW, QZ, BL, AL, IG, and LL acquired the data. GY and LW analyzed and interpreted the data. GY and JS drafted the manuscript. GY, XC, and HJ critically revised the article. SZ approved the final version of the manuscript on behalf of all authors. DZ, LW, and JS statistically analyzed the data. SZ supervised the study.

## Supporting information

Figure S1

Figure S2

Table S1

## Data Availability

The data that support the findings of this study are available from the corresponding author upon reasonable request.
