# Supplementary figures and images for: Left ventricular ejection fraction as an independent predictor of poor outcome in acute intracerebral hemorrhage
Source: Brain Behav. 2020 Jun 9;10(7):e01643. doi: 10.1002/brb3.1643 (PMC7375101; doi:10.1002/brb3.1643)

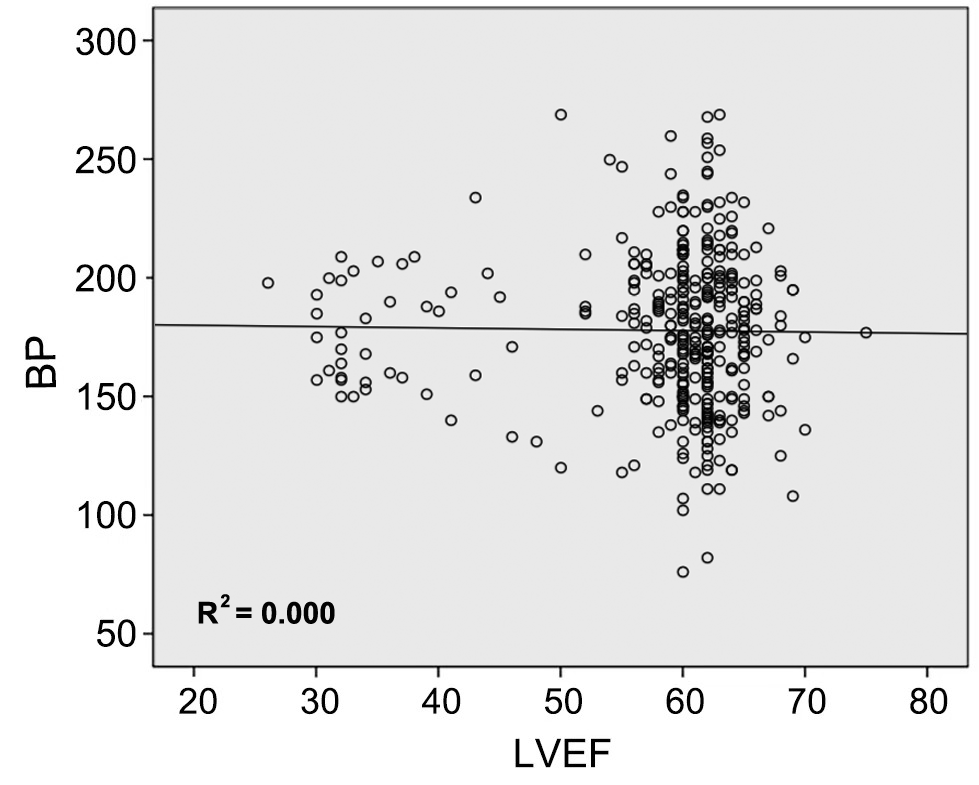

Supplement: Supplementary file 1 — Figure S1 [file BRB3-10-e01643-s001.tif]

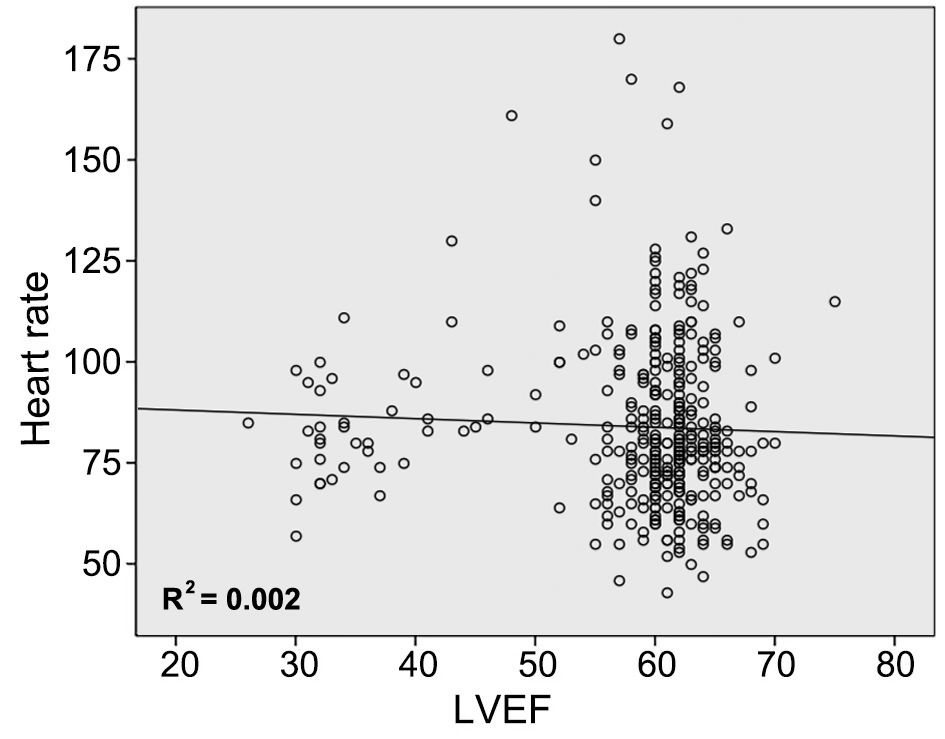

Supplement: Supplementary file 2 — Figure S2 [file BRB3-10-e01643-s002.tif]
