# Supplementary material for: Left ventricular ejection fraction as an independent predictor of poor outcome in acute intracerebral hemorrhage
Source: Brain Behav. 2020 Jun 9;10(7):e01643. doi: 10.1002/brb3.1643 (PMC7375101; doi:10.1002/brb3.1643)
Supplement: Supplementary file 3 — Table S1 [file BRB3-10-e01643-s003.docx]

Supplemental Table 1 Characteristics of ICH patients according to LVEF on admission (n = 364).

| **Characteristics** | **LVEF ≥ 50% (n=325)** | **LVEF < 50%**  **(n=39)** | **P Value** |
| --- | --- | --- | --- |
| Age, y | 60.6 ± 13.3 | 64.7 ± 13.4 | 0.072 |
| Sex (male) | 216 (66.5) | 24 (61.5) | 0.540 |
| History of Hypertension | 228 (70.2) | 24 (61.5) | 0.271 |
| History of Diabetes | 57 (17.5) | 4 (10.3) | 0.250 |
| CAD | 55 (16.9) | 5 (12.8) | 0.308 |
| History of atrial fibrillation | 26 (8.0) | 5 (12.8) | 0.514 |
| First systolic BP, mm Hg | 178.1 ± 33.1 | 176.3 ± 24.2 | 0.746 |
| Heart Rate | 83.7 ± 20.7 | 86.9 ± 18.4 | 0.362 |
| Temperature, ℃ | 36.6 ± 0.5 | 36.7 ± 0.6 | 0.608 |
| Admit GCS | 13 (9-15) | 15 (13-15) | 0.036 |
| Admit NIHSS | 10 (4-19) | 8 (3-12) | 0.205 |
| Anticoagulant | 59 (18.2) | 6 (15.4) | 0.670 |
| Antiplatelet | 71 (21.8) | 7 (17.9) | 0.575 |
| Initial hematoma volume, ml | 14.2 (6.2-33.6) | 11.4 (4.0-29.5) | 0.211 |
| IVH | 113 (34.8) | 14 (35.9) | 0.589 |
| Location of ICH |  |  | 0.766 |
| Lobar | 44 (13.5) | 4 (10.3) |  |
| Deep | 210 (64.6) | 25 (64.1) |  |
| Infratentorial | 37 (11.4) | 3 (7.7) |  |
| Midline shift, mm | 3.5 ± 3.9 | 3.3 ± 3.5 | 0.812 |
| Admission HCT, % | 42.2 (38.6-45.3) | 39.8 (37.7-42.6) | 0.083 |
| Admission platelet count, K/uL | 223.7 (185.2-265.4) | 204.4 (170.3-252.6) | 0.360 |
| Admission INR | 1.1 (1.0-1.1) | 1.1 (1.0-1.2) | 0.169 |
| Glucose, mmol/l | 7.1 (5.8-9.7) | 6.9 (6.3-8.3) | 0.234 |
| Surgery | 94 (28.9) | 9 (23.1) | 0.444 |

Abbreviations: BP = blood pressure; CAD = coronary artery disease; GCS = Glasgow Coma Scale; HCT = hematocrit; ICH = intracerebral hemorrhage; INR = international normalized ratio; IVH = intraventricular hemorrhage on presentation; LVEF = left ventricular ejection fraction; mRS = modified Rankin Scale; NIHSS = NIH Stroke Scale.

Values are n (%), mean ± SD, or median (interquartile range).
